# Burden of Cervical Cancer in the Eastern Mediterranean Region During the Years 2000 and 2017: Retrospective Data Analysis of the Global Burden of Disease Study

**DOI:** 10.2196/22160

**Published:** 2021-05-12

**Authors:** Fereshteh Safaeian, Shidrokh Ghaemimood, Ziad El-Khatib, Sahba Enayati, Roksana Mirkazemi, Bruce Reeder

**Affiliations:** 1 Public Health Graduate Studies The Bahá'í Institute for Higher Education Tehran Iran; 2 World Health Programme Université du Québec en Abitibi-Témiscamingue Québec, QC Canada; 3 Internal Medicine and Cardiology Kompetenzcenter Gesundheit, St Stephan Wels Austria; 4 Department of Community Health and Epidemiology University of Saskatchewan Saskatoon, SK Canada

**Keywords:** cervical cancer, Eastern Mediterranean Region, burden of disease, cancer, burden, inequality, mortality, preventable disease

## Abstract

**Background:**

Cervical cancer is a growing health concern, especially in resource-limited settings.

**Objective:**

The objective of this study was to assess the burden of cervical cancer mortality and disability-adjusted life years (DALYs) in the Eastern Mediterranean Region (EMR) and globally between the years 2000 and 2017 by using a pooled data analysis approach.

**Methods:**

We used an ecological approach at the country level. This included extracting data from publicly available databases and linking them together in the following 3 steps: (1) extraction of data from the Global Burden of Disease (GBD) study in the years 2000 and 2017, (2) categorization of EMR countries according to the World Bank gross domestic product per capita, and (3) linking age-specific population data from the Population Statistics Division of the United Nations (20-29 years, 30-49 years, and >50 years) and GBD’s data with gross national income per capita and globally extracted data, including cervical cancer mortality and DALY numbers and rates per country. The cervical cancer mortality rate was provided by the GBD study using the following formula: number of cervical cancer deaths × 100,000/female population in the respective age group.

**Results:**

The absolute number of deaths due to cervical cancer increased from the year 2000 (n=6326) to the year 2017 (n=8537) in the EMR; however, the mortality rate due to this disease decreased from the year 2000 (2.7 per 100,000) to the year 2017 (2.5 per 100,000). According to age-specific data, the age group ≥50 years showed the highest mortality rate in both EMR countries and globally, and the age group of 20-29 years showed the lowest mortality rate both globally and in the EMR countries. Further, the rates of cervical cancer DALYs in the EMR were lower compared to the global rates (2.7 vs 6.8 in 2000 and 2.5 vs 6.8 in 2017 for mortality rate per 100,000; 95.8 vs 222.2 in 2000 and 86.3 vs 211.8 in 2017 for DALY rate per 100,000; respectively). However, the relative difference in the number of DALYs due to cervical cancer between the year 2000 and year 2017 in the EMR was higher than that reported globally (34.9 vs 24.0 for the number of deaths and 23.5 vs 18.1 for the number of DALYs, respectively).

**Conclusions:**

We found an increase in the burden of cervical cancer in the EMR as per the data on the absolute number of deaths and DALYs. Further, we found that the health care system has an increased number of cases to deal with, despite the decrease in the absolute number of deaths and DALYs. Cervical cancer is preventable if human papilloma vaccination is taken and early screening is performed. Therefore, we recommend identifying effective vaccination programs and interventions to reduce the burden of this disease.

## Introduction

Globally, cervical cancer is one of the leading causes of mortality in women [1] mainly due to human papillomavirus (HPV) infection, smoking, and other risk factors [2]. Although cervical cancer is highly preventable [3], it contributes to the death of 260,000 women each year, of which nearly 85% occur in low- and middle-income countries (LMICs) [4]. Global cervical cancer incidence has increased from 378,000 cases in 1980 to 454,000 cases in 2010—an annual rate increase of 0.6% [5]. New cases of cervical cancer occur in all age groups more often in LMICs than in high-income countries (HICs), where 46,000 persons out of 200,000 persons are in the age range of 15-49 years [5]. Additionally, there is a link between cervical cancer and global inequalities, especially in the LMICs [6,7]. The cervical cancer mortality rate has decreased in HICs owing to effective screening, access to treatment, and vaccinations. Currently, the major burden of cervical cancer is present in the LMICs [8].

The Eastern Mediterranean Region (EMR) consists of 22 countries in the Middle East, North Africa, the Horn of Africa, and Central Asia. This region includes some of the greatest social inequalities within countries and between countries in the world [9]. Health disparities in this region are paralleled with disparities in socioeconomic development [10]. The profile of cancer prevalence in the EMR in 2017 showed that the highest proportions of cervical cancer were found in North Africa and the Horn of Africa [11]. In these countries, nationwide programs of cervical cancer screening do not exist or are based on a limited opportunistic cytology-based screening, which often lacks quality assurance [12].

This study examined the burden of cervical cancer and potential differences within the countries in the EMR in relation to the level of national economic development and age of the patients. The objective of this study was to assess the burden of cervical cancer mortality and disability-adjusted life years (DALYs) as the number of years lost due to disability or early death because of cervical cancer in countries of the EMR and globally. The following research questions were investigated:

1. What is the pattern of mortality and DALYs due to cervical cancer in the EMR and globally? Further, what changes in this pattern have occurred between the years 2000 and 2017?
2. Do these rates differ by the level of national economic development and age of the population?

## Methods

### Data Sources

This was an ecological study that was conducted at the country level. Three publicly available databases were linked together in a three-step process. The first step consisted of extracting cervical cancer data at the country level from the Global Burden of Disease (GBD) study in 2000 and 2017 [13] to provide the numbers and rates of cervical cancer mortality as well as the numbers and rates of DALYs. We chose the data from the years 2000 and 2017 as the most recent data available from the cancer registry system.

### Measures and Analyses

The EMR consists of the following 22 countries: Afghanistan, Bahrain, Djibouti, Egypt, Iraq, Iran, Jordan, Kuwait, Lebanon, Libya, Morocco, Oman, Pakistan, Palestine (West Bank and Gaza), Qatar, Saudi Arabia, Somalia, Sudan, Syria, Tunisia, and the United Arab Emirates [14]. The cervical cancer mortality of 122 countries was expressed as the overall mortality and DALYs (number and rates) in the GBD study. As the countries vary significantly in terms of their levels of economic development, they were stratified, in the second step, to 3 standard world economy groups according to the World Bank definition based on the gross national income per capita in 2017: HICs at >US $12,056, MICs at US $996-12,055, and low-income countries (LICs) at <US $995 [14]. The LICs are Afghanistan and Somalia. The MICs are Djibouti, Egypt, Iraq, Iran, Jordan, Lebanon, Libya, Morocco, Pakistan, Palestine (West Bank and Gaza), Sudan, Syria, Yemen, and Tunisia. The HICs are Bahrain, Saudi Arabia, Kuwait, Oman, Qatar, and the United Arab Emirates [14]. The third step included extracting age-specific population data (rate and frequency) from the Population Statistics Division of the United Nations [15]. This was necessary as the GBD study data were different from those of the Population Statistics Division and they provide no rates at regional or income level.

Cervical cancer mortality rates and DALY rates per 100,000 persons were extracted from the GBD database for people older than 20 years at the country level in the years 2000 and 2017 and then combined for an EMR average (n=22) and global average of EMR plus all other countries (n=122). The mortality rate formula was as follows: cervical cancer deaths (n) × 100,000/female population in the respective age group. The results were then stratified by the 3 income levels (high, middle, low) and 3 age groups (20-29 years, 30-49 years, ≥50 years). The burden of cervical cancer mortality was presented through the compilation of 3 variables: (1) average mortality rates per 100,000 with minimum and maximum values to provide the range as a measure of spread for cervical cancer mortality and mortality from other diseases, (2) DALYs as a measure of total burden, whereby 1 DALY represents 1 healthy year of life lost to cervical cancer; the GBD study did estimate DALYs by summing the fatal burden (years of life lost) and nonfatal burden (years of life with disability) due to cervical cancer [16], and (3) relative difference (%) and absolute difference between years 2000 and 2017 to show the comparative changes in deaths and DALY numbers. The absolute difference equals the difference between the 2 comparing numbers of years 2000 and 2017. The relative difference is equal to the value of the absolute difference divided by the value in the year 2000, and then we multiplied it by 100 [17].

## Results

The rates of cervical cancer mortality and DALYs in the EMR were lower than the global rates; however, the relative difference in the number of deaths and DALYs due to cervical cancer between the years 2000 and 2017 in the EMR was higher than that of the global data. Further, according to age-specific data, the age group of ≥50 years had the highest mortality rates in both EMR countries and globally and the age group of 20-29 years had the lowest mortality rate both globally and in the EMR countries. The EMR average mortality rate due to cervical cancer was lower than the global average for both reference years and all 3 income levels (Table 1). Within income levels, the differences between EMR countries and the global average is the most remarkable in the HICs. During the period 2000-2017, there was a decline in the mortality rate in the LMICs, both globally and in the EMR. Although there was a decline in the average mortality rate in the EMR, there was an increase in the mortality rates in Egypt, Iran, Libya, Morocco, Syria, Tunisia, and Yemen. Further, although the mortality rate in the EMR according to the 3 income levels compared to the global mortality rate was lower, the relative difference in the number of deaths compared to the global relative difference in the number of deaths was higher (Table 1). The relative change in the number of deaths due to cervical cancer in the EMR for all income levels over the course of 2000 and 2017 was greater than that seen globally (relative difference 34.9 vs 24.0, respectively) (Table 1). In the EMR, the HICs showed the greatest relative increase in the number of deaths (85.5%), and among LICs, Afghanistan had greater relative increase compared to Somalia. Among MICs, Iran had the greatest relative difference and Iraq had the lowest; among the HICs, United Arab Emirates showed the greatest relative increase and Bahrain the lowest (Table 1).

**Table 1:** Cervical cancer mortality among women older than 20 years in the Eastern Mediterranean Region by country and income level compared to the global average in the years 2000 and 2017.

Group by income level, country		Cervical cancer mortality rate per 100,000 women in year 2000, mean (min-max)	Cervical cancer deaths (n) in year 2000	Cervical cancer mortality rate per 100,000 women in year 2017, mean (min-max)	Cervical cancer deaths (n) in year 2017	Relative difference (%) in the number of deaths between years 2000 and 2017^a^	Absolute difference in the number of deaths between years 2000 and 2017^b^
Low-income countries globally		12.4 (9.1-16.2)	26,884	10.0 (7.1-13.6)	33,591	24.9	6707
**Low-income countries in the EMR^c^**		10.7 (6.3-15.0)	1448	7.9 (4.7-11.5)	1930	33.3	482
	Afghanistan	5.3 (1.9-7.9)	468	3.9 (1.7-5.7)	631	34.9	163
	Somalia	20.9 (14.7-28.3)	980	15.8 (10.6-22.9)	1299	32.5	319
Middle-income countries globally		6.5 (5.7-7.7)	149,272	6.7 (5.4-8.0)	192,643	29.1	43,370
**Middle-income countries in the EMR**		2.3 (1.9-2.8)	4755	2.2 (1.6-2.8)	6380	34.2	1625
	Djibouti	16.0 (10.6-22.9)	48	13.8 (8.7-21.8)	72	50.4	24
	Egypt	0.9 (0.8-1.0)	292	1.0 (0.8-1.2)	449	53.6	157
	Iran	1.2 (1.0-1.3)	397	1.8 (1.4-1.9)	726	82.7	329
	Iraq	1.5 (1.0-2.1)	186	0.8 (0.7-0.9)	168	–9.4	–18
	Jordan	1.5 (1.2-1.9)	35	1.0 (0.8-1.3)	48	37.2	13
	Lebanon	2.4 (2.0-2.8)	63	2.0 (1.7-2.3)	82	31.2	20
	Libya	2.5 (2.0-3.0)	61	3.2 (2.4-4.1)	106	74.4	45
	Morocco	5.5 (4.5-6.4)	823	5.7 (4.1-7.5)	1011	22.8	187
	Pakistan	3.3 (2.8-3.8)	2213	2.8 (2.0-3.8)	2886	30.4	673
	Palestine	1.2 (1.0-1.4)	18	1.2 (0.9-1.4)	28	56.2	10
	Syria	0.9 (0.8-1.1)	73	1.1 (0.8-1.4)	98	34.1	25
	Tunisia	2.4 (2.0-2.9)	119	2.5 (1.8-3.4)	145	22.5	27
	Sudan	1.8 (1.0-2.6)	242	1.4 (0.9-2.0)	281	15.9	39
	Yemen	0.9 (0.8-1.1)	185	1.9 (1.2-2.6)	279	50.8	94
High-income countries globally		6.0 (5.8-6.2)	32,475	5.4 (5.0-5.9)	32,444	–0.1	–31
**High-income countries in the EMR**		1.0 (0.8-1.2)	123	1.0 (0.8-1.4)	227	85.5	105
	Bahrain	1.9 (1.7-2.3)	5	1.5 (1.2-1.8)	8	51.1	3
	Kuwait	1.1 (1.0-1.1)	8	0.7 (0.6-0.8)	14	59.5	5
	Oman	1.7 (1.2-2.2)	17	1.5 (1.1-1.8)	23	35.9	6
	Qatar	1.4 (1.1-1.8)	3	1.0 (0.7-1.2)	6	122.9	4
	Saudi Arabia	0.7 (0.6-0.9)	68	0.8 (0.6-1.1)	117	71.1	49
	United Arab Emirates	2.0 (1.5-2.4)	21	2.3 (1.7-3.1)	60	187.2	39
Global average (122 countries)		6.8 (6.4-7.6)	208,631	6.8 (6.3-7.1)	258,678	24.0	50,047
EMR average (22 countries)		2.7 (2.1-3.4)	6326	2.5 (1.8-3.4)	8537	34.9	2211

^a^Relative difference (%) in the number of deaths = (absolute difference in the number of deaths/number of cervical cancer deaths in 2000)*100.

^b^Absolute difference in the number of deaths = number of cervical cancer deaths in 2017 – number of cervical cancer deaths in 2000.

^c^EMR: Eastern Mediterranean Region.

DALYs due to cervical cancer by income level globally and in EMR countries have been shown in Table 2. The LICs had the highest rates of DALYs both globally and in the EMR, with rates in the EMR exceeding those seen globally. Somalia had the highest rate of 761.5 DALYs in 2000 and 554.5 DALYs in 2017. The MICs in the EMR had a lower DALY rate than other countries globally in both reference years. Within this income level, Djibouti had the highest rate of 585.7 DALYs in 2000 and 497.6 DALYs in 2017. Globally, HICs showed a decline in the DALYs in 2017 whereas HICs in the EMR showed an increase in 2017. Within this income level, Emirates and Bahrain had the highest rate of 65.6 DALYs per 100,000 in the year 2000 and Emirates had the highest rate of 81.9 DALYs per 100,000 in the year 2017 (Table 2). The relative difference in the number of DALYs in the EMR for the 3 income levels was higher than that in the DALYs globally (Table 2). Among LICs in the EMR, Afghanistan had the highest relative difference in the number of DALYs and among the MICs in the EMR, Libya had the highest relative difference and Iraq had the lowest relative difference in the number of DALYs. Among the HICs in the EMR, Emirates had the highest relative difference in the number of DALYs and Oman had the lowest relative difference in the number of DALYs (Table 2).

**Table 2:** Disability-adjusted life years data due to cervical cancer among women older than 20 years in the Eastern Mediterranean Region by country and income level compared to the global average in the years 2000 and 2017.

Group by income level, country		Cervical cancer DALY^a^ rate per 100,000 women in year 2000, mean (min-max)	DALY number in year 2000	Cervical cancer DALY rate per 100,000 women in year 2017, mean (min-max)^c^	DALY number in year 2017	Relative difference (%) in the number of DALYs between years 2000 and 2017^b^	Absolute difference in the number of DALYs between years 2000 and 2017^c^
Low-income countries globally		435.3 (312.8-566.6)	941,128	343.9 (239.4-472.8)	1,157,715	23.0	216,587
**Low-income countries in the EMR^d^**		388.8 (227.8-553.0)	52,554	287.0 (162.8-424.4)	69,812	32.8	17,258
	Afghanistan	191.2 (65.2-296.4)	16,895	150.6 (64.5-227.1)	24,265	43.6	7371
	Somalia	761.5 (534.7-1037.1)	35,659	554.5 (355.7-811.5)	45,546	27.7	9887
Middle-income countries globally		217.4 (187.6-260.8)	5,003,701	213.0 (167.5-255.4)	6,086,292	21.6	1,082,592
**Middle-income countries in the EMR**		80.5 (64.7-97.8)	165,567	73.3 (52.9-98.2)	216,480	30.8	50,913
	Djibouti	585.7 (389.7-840.8)	1754	497.6 (308.6-795.5)	2598	48.1	844
	Egypt	31.2 (27.1-36.3)	10,191	32.3 (25.7-40.2)	15,065	47.8	4875
	Iran	39.5 (33.3-43.3)	13,058	51.8 (41.6-55.6)	20,993	60.8	7936
	Iraq	51.8 (32.5-74.8)	6619	26.8 (22.2-32.4)	5636	–14.9	–983
	Jordan	52.2 (42.9-64.8)	1223	29.8 (22.9-39.4)	1482	21.2	259
	Lebanon	77.4 (63.4-91.9)	2043	59.3 (49.4-72.1)	2505	22.6	461
	Libya	85.0 (68.0-104.7)	2092	112.2 (81.8-145.0)	3758	79.7	1667
	Morocco	182.7 (147.2-215.1)	27,265	181.1 (131.0-239.0)	31,984	17.3	4720
	Pakistan	118.4 (100.9-139.9)	80,013	100.8 (71.4-141.5)	105,213	31.5	25,200
	Palestine	36.6 (29.3-42.7)	545	34.7 (26.2-41.2)	825	51.5	280
	Syria	32.0 (26.8-39.2)	2592	34.3 (25.1-44.2)	3051	17.7	459
	Tunisia	73.4 (59.7-87.3)	3604	71.0 (50.7-94.7)	4075	13.1	471
	Sudan	60.8 (31.9-90.8)	8142	48.5 (28.6-69.5)	9644	18.5	1502
	Yemen	70.4 (38.8-105.6)	6426	64.1 (39.1-92.6)	9648	50.1	3222
High-income countries globally		158.4 (152.4-164.8)	854,589	131.7 (119.7-144.8)	787,560	–7.8	–67,028
**High-income countries in the EMR**		32.8 (26.7-41.1)	4193	38.0 (28.7-50.3)	8274	97.3	4081
	Bahrain	65.6 (57.3-78.7)	183	46.8 (39.2-57.1)	261	42.9	78
	Kuwait	39.3 (35.7-43.2)	315	26.5 (22.9-30.5)	515	63.5	200
	Oman	58.9 (42.9-78.7)	598	50.6 (38.1-64.3)	790	32.2	193
	Qatar	47.7 (37.6-61.5)	99	33.8 (25.5-44.0)	228	131.7	130
	Saudi Arabia	24.4 (20.2-30.7)	2302	30.1 (22.8-40.4)	4348	88.9	2046
	United Arab Emirates	65.6 (51.8-82.0)	697	81.9 (58.6-111.6)	2132	205.8	1435
Global average (122 countries)		222.2 (206.2-245.9)	6,799,418	211.8 (197.8-220.8)	8,031,567	18.1	1,232,149
EMR average (22 countries)		95.8 (72.1-121.2)	222,314	86.3 (59.2-118.4)	294,566	32.5	72,252

^a^DALY: disability-adjusted life year.

^b^Relative difference (%) in the number of DALYs = (absolute difference in the number of DALYs/number of cervical cancer DALYs in 2000)*100.

^c^Absolute difference in the number of DALYs = number of cervical cancer DALYs in 2017 – number of cervical cancer DALYs in 2000.

^d^EMR: Eastern Mediterranean Region.

The age group of ≥50 years had the highest mortality rate in both EMR countries and globally and the age group of 20-29 years showed the lowest mortality rate both globally and in the EMR countries. Among EMR countries, Somalia and Djibouti showed the highest mortality rate in all the age groups. Kuwait had the lowest mortality rate for the age group of ≥50 years (5.9 per 100,000 in 2000 and 3.3 per 100,000 in 2017) and Qatar had the lowest mortality rate in the age group of 30-49 years (1.5 per 100,000 in 2000 and 0.7 per 100,000 in 2017). Oman had the lowest mortality rate of 0 per 100,000 in 2017 for the age group of 20-29 years (Table 3).

The relative difference in the number of deaths between 2000 and 2017 in EMR-LICs for age groups 20-29 years and 30-49 years was higher than the global difference (12.8% and 49.6% vs 4.7% and 21.9, respectively), but it was lower for the age group >50 years (25.1% vs 27.3%) (Table 3). In MICs, this variable in EMR countries was lower than that seen globally for the age groups 20-29 years and 30-49 years (13.5% and 27.4% vs –24.4% and 4.3%, respectively), but it was higher for the age group >50 years (39.0% vs 43.7%). The relative difference in the number of deaths in EMR-HICs for the 3 age groups was higher than that reported globally (25.6%, 110%, and 74.8% vs –16.5%, –26.9%, and 6.7%, respectively). Among the EMR countries, the United Arab Emirates and Saudi Arabia showed an increase in the relative difference in the number of deaths in 2017. Further, in the EMR-MICs, Egypt, Iran, Libya, and Syria showed an increase in the relative difference in the number of deaths in 2017, while LICs showed a decrease in the relative difference in 2017 (Table 3).

**Table 3:** Cervical cancer mortality rates among women older than 20 years in the Eastern Mediterranean Region by age, country, and income level compared to the global average in the years 2000 and 2017.

Group by income level, country		Average cervical cancer mortality rate per 100,000 women by age range						Relative difference (%) in the number of deaths between years 2000 and 2017^a^		
		Year 2000, mean (min-max)			Year 2017, mean (min-max)					
		20-29 years	30-49 years	≥50 years	20-29 years	30-49 years	≥50 years	20-29 years	30-49 years	≥50 years
Low-income countries globally		2.5 (1.3-4.2)	53.0 (32.3-79.4)	94.8 (67.8-127.6)	1.6 (0.8-2.9)	38.2 (22.6-59.8)	77.4 (53.7-107.7)	4.7	21.9	27.3
**Low-income countries in the EMR^b^**		2.0 (0.8-3.7)	57.8 (28.7-96.4)	72.2 (42.6-101.8)	1.5 (0.7-2.9)	34.6 (16.1-59.7)	72.9 (44.5-106.7)	12.8	49.6	25.1
	Afghanistan	1.3 (0.4-2.7)	31.1 (8.6-57.8)	34.8 (14.4-52.0)	1.1 (0.4-2.1)	20.6 (7.0-36.8)	31.9 (16.2-45.6	19.7	105.0	–0.1
	Somalia	3.3 (1.7-5.7)	90.8 (53.5-143.2)	153.2 (108.4-208.4)	2.4 (1.2-4.5)	65.6 (36.6-109.1)	135.3 (90.3-201.5)	7.1	26.1	37.5
Middle-income countries globally		1.0 (0.7-1.4)	16.9 (13.5-21.3)	31.8 (27.7-38.4)	0.6 (0.4-0.9)	13.0 (9.5-16.6)	26.6 (21.1-32.0)	–24.4	4.3	43.7
**Middle-income countries in the EMR**		0.5 (0.3-0.8)	7.7 (4.9-11.3)	15.9 (12.3-19.7)	0.4 (0.2-0.8)	5.7 (3.4-8.9)	13.3 (9.7-17.7)	13.5	27.4	39.0
	Djibouti	2.1 (1.1-3.7)	63.2 (37.1-101.8)	113.9 (75.3-159.5)	1.3 (0.6-2.4)	41.8 (23.2-71.6)	78.7 (50.3-121.1)	8.1	49.1	53.1
	Egypt	0.1 (0.1-0.2)	2.8 (1.9-4.0)	6.3 (5.1-7.6)	0.1 (0.1-0.2)	2.6 (1.6-3.8)	6.7 (5.1-8.6)	34.2	35.8	64.6
	Iran	0.2 (0.2-0.3)	3.3 (2.7-3.8)	8.5 (7.2-9.5	0.2 (0.1-0.2)	2.5 (2.0-2.8)	10.2 (7.9-11.1)	–21.4	37.3	109.0
	Iraq	0.3 (0.5-0.1)	6.3 (3.1-10.3)	11.9 (9.0-16.4)	0.1 (0.1-0.2)	2.3 (3.3-1.6)	5.6 (4.6-6.7)	–23.3	–24.1	0.6
	Jordan	0.2 (0.1-0.4)	5.8 (3.9-8.2)	12.4 (10.0-16.6)	0.1 (0.0-0.1)	1.9 (1.2-2.9)	7.5 (5.7-10.3)	–26.5	5.2	56.9
	Lebanon	0.3 (0.2-0.5)	7.2 (4.7-10.5)	15.4 (12.3-19.0)	0.2 (0.1-0.3)	4.0 (2.6-5.8)	12.2 (9.6-15.2)	2.8	20.4	36.6
	Libya	0.4 (0.2-0.7)	8.8 (5.7-12.9)	16.9 (13.1-22.2)	0.3 (0.2-0.6)	7.8 (4.7-11.9)	15.4 (11.3-20.6)	–0.3	97.8	65.9
	Morocco	0.5 (0.3-0.9)	15.5 (9.9-22.1)	30.6 (23.5-37.0)	0.4 (0.2-0.7)	11.1 (6.6-17.3)	23.8 (17.3-31.2)	–12.6	1.8	32.8
	Pakistan	1.1 (0.6-1.7)	11.8 (7.8-17.0)	20.8 (16.5-25.3)	0.8 (0.4-1.6)	9.0 (5.3-14.5)	17.1 (12.5-24.2)	24.3	37.6	27.1
	Palestine	0.2 (0.1-0.2)	3.8 (2.5-5.4)	13.8 (11.0-16.5)	0.1 (0.1-0.2)	2.6 (1.7-3.8)	12.1 (8.8-14.7)	18.0	40.8	62.6
	Syria	0.2 (0.1-0.3)	3.6 (2.5-5.1)	7.0 (5.8-8.9)	0.1 (0.1-0.2)	2.2 (1.4-3.6)	6.5 (5.0-8.1)	–48.4	–6.4	63.6
	Tunisia	0.2 (0.1-0.3)	5.4 (3.5-7.7)	14.3 (11.6-18.0)	0.1 (0.1-0.2)	3.4 (2.0-5.3)	11.2 (8.0-15.3)	–33.2	–10.9	35.6
	Sudan	0.4 (0.1-0.7)	7.2 (2.9-12.5)	14.6 (9.2-22.2)	0.2 (0.1-0.4)	5.2 (2.4-8.7)	11.3 (7.6-15.9)	–0.9	22.1	13.5
	Yemen	0.4 (0.2-0.7)	9.5 (4.1-16.3)	18.8 (11.1-28.4)	0.3 (0.1-0.5)	7.1 (3.4-11.9)	15.5 (10.6-22.1)	30.3	49.0	52.9
High-income countries globally		0.4 (0.3-0.4)	8.2 (7.4-9.1)	19.5 (18.6-20.5)	0.3 (0.2-0.4)	5.6 (4.7-6.7)	14.9 (13.4-16.4)	–16.5	–26.9	6.7
**High-income countries in the EMR**		0.1 (0.1-0.2)	2.3 (1.6-3.4)	8.4 (6.7-10.7)	0.1 (0.0-0.1)	1.7 (1.0-2.6)	7.2 (5.4-9.2)	25.6	110.0	74.8
	Bahrain	0.2 (0.1-0.4)	3.3 (2.3-4.7)	17.4 (14.4-22.6)	0.1 (0.1-0.2)	1.2 (0.8-1.7)	9.6 (7.7-12.3)	–6.8	7.2	75.5
	Kuwait	0.1 (0.1-0.2)	2.5 (1.9-3.3)	5.9 (5.2-6.6)	0.1 (0.0-0.1)	1.5 (1.1-2.0)	3.3 (2.9-3.8)	–0.5	67.4	57.5
	Oman	0.2 (0.1-0.4)	5.3 (3.3-8.1)	14.2 (10.9-18.8)	0.1 (0.0-0.1)	2.4 (1.5-3.8)	10.2 (7.6-13.0)	15.8	25.5	43.9
	Qatar	0.1 (0.0-0.1)	1.5 (0.9-2.3)	15.2 (19.4-11.8)	0.0 (0.0-0.1)	0.7 (0.4-1.1)	8.0 (6.0-10.4)	184.6	125.6	120.7
	Saudi Arabia	0.1 (0.1-0.1)	2.0 (1.4-2.8)	6.3 (5.2-7.8)	0.1 (0.0-0.1)	1.7 (1.1-2.6)	5.3 (4.1-6.6)	38.5	109.5	51.2
	United Arab Emirates	0.1 (0.1-0.2)	2.5 (1.6-3.8)	26.6 (19.7-33.7)	0.1 (0.1-0.2)	1.7 (1.0-2.7)	21.2 (15.1-27.8)	2.3	256.5	165.6
Global average (122 countries)		1.0 (0.9-1.1)	16.7 (15.3-18.5)	29.9 (28.4-32.8)	0.7 (0.6-0.7)	13.3 (12.1-14.0)	24.9 (23.3-25.9)	–19.1	3.8	35.0
EMR average (22 countries)		0.6 (0.3-1.0)	9.2 (5.6-14.0)	18.9 (13.8-24.2)	0.4 (0.2-0.8)	6.7 (3.8-10.7)	15.6 (11.0-21.1)	13.5	34.5	36.6

^a^Relative difference (%) in the number of deaths = ([number of cervical cancer deaths in 2017 – number of cervical cancer deaths in 2000]/number of cervical cancer deaths in 2000)*100.

^b^EMR: Eastern Mediterranean Region.

Both globally and in the EMR, DALYs declined for all age groups in the period of 2000-2017. The global average of DALYs for the 3 age groups was higher than the EMR average in both the reference years, and the age group of 30-49 years showed the highest number of DALYs (Table 4). The age group of 30-49 years showed the highest rate of DALYs both globally and in the EMR and for the 3 income levels. HICs in the EMR was the exception in this age group as they showed a lower rate of DALYs compared to the age group of ≥50 years. LICs, both globally and in the EMR, showed a decline in the DALYs for the 3 age groups in 2017 compared to 2000. Somalia had the highest number of DALYs for all 3 age groups and both reference years. MICs in the EMR showed lower rates of DALYs for the 3 age groups and in both reference years. Djibouti had the highest rates of DALYs for all age groups and in both reference years. Egypt had the lowest rates of DALYs for all 3 age groups in 2000 and for the age group ≥50 years in 2017. Jordan had the lowest number of DALYs for the age groups of 20-29 years and 30-49 years in 2017 (Table 4). The distribution of the relative difference in the number of DALYs for the different age groups showed that the DALYs of EMR-LICs were higher than those of the global countries for the age groups of 20-29 years and 30-49 years (14% and 50.6% vs 5.5% and 21.8%, respectively). Further, it was lower for the age group >50 years (18.2% vs 26%, respectively). Afghanistan had the highest relative difference in the number of DALYs of 95.0% for the age group of 30-49 years (Table 4). EMR-MICs had higher relative difference in the number of DALYs than the global MICs for all 3 age groups (Table 4). The age group of 20-29 years in Egypt had the highest relative difference in the number of DALYs of 35.7%, and the age group of 30-49 years in Libya had the highest relative difference of 95.9%. For the age group >50 years, Iran had the highest relative difference in the number of DALYs of 98.0%. Finally, EMR-HICs had higher relative difference in the number of DALYs than global HICs for all 3 age groups (28.1%, 111.6%, and 90.6% vs –15.4%, –26.2%, and 3.4%, respectively). Among EMR-HICs, the age group of 20-29 years in Qatar had the highest relative difference in the number of DALYs of 192.8% and age groups of 30-49 years and >50 years in Emirates had the highest relative difference of 258.9% and 185.6%, respectively (Table 4).

**Table 4:** Disability-adjusted life years data due to cervical cancer among women older than 20 years in the Eastern Mediterranean Region by age, country, and income level compared to the global average in the years 2000 and 2017.

Group by income level, country		Average cervical cancer DALY^a^ rate per 100,000 women by age range						Relative difference (%) in number of DALYs ^b^ (2000 vs 2017)		
		Year 2000, mean (min-max)			Year 2017, mean (min-max)					
		20-29 years	30-49 years	≥50 years	20-29 years	30-49 years	≥50 years	20-29 years	30-49 years	≥50 years
Low-income countries globally		161.5 (85.5-268.9)	2473.0 (1496.9-3715.7)	2062.9 (1466.6-2779.8)	106.7 (54.8-188.5)	1783.2 (1051.3-2807.4)	1641.5 (1128.3-2294.7)	5.5	21.8	26.0
**Low-income countries in the EMR^c^**		128.1 (53.6-235.7)	2637.7 (1294.6-4425.8)	1689.7 (986.3-2398.2)	97.3 (43.3-187.5)	1628.2 (754.6-2815.3)	1617.3 (972.9-2364.2)	14.0	50.6	18.2
	Afghanistan	85.8 (25.8-169.6)	1456.7 (402.8-2719.2)	783.9 (300.6-1183.2)	69.9 (25.8-136.6)	971.6 (331.2-1749.2)	697.3 (330.0-1001.0)	21.9	95.0	–1.8
	Somalia	213.8 (109.5-370.0)	4186.9 (2458.3-6609.7)	3555.1 (2483.6-4884.5)	152.5 (77.2-294.0)	3022.9 (1679.8-5033.9)	3006.5 (1978.5-4457.5)	7.6	30.4	27.3
Middle-income countries globally		65.6 (47.3-88.9)	801.3 (638.8-1011.8)	642.6 (555.4-778.8)	43.1 (29.8-62.3)	628.7 (447.7-838.0)	532.0 (412.6-643.9)	–81.4	–86.1	17.6
**Middle-income countries in the EMR**		34.5 (19.7-55.9)	368.1 (232.7-542.7)	326.8 (249.3-407.8)	26.8 (13.7-50.3)	272.3 (162.1-429.7)	259.5 (185.7-345.1)	14.6	28.3	36.0
	Djibouti	135.4 (69.1-237.0)	2907.3 (1707.0-4709.3)	2569.5 (1675.6-3674.6)	82.2 (39.0-155.3)	1940.6 (1076.8-3330.4)	1714.6 (1082.4-2677.9)	8.7	50.9	49.2
	Egypt	8.9 (5.1-14.4)	134.6 (90.5-193.0)	120.5 (97.7-146.7)	8.3 (4.4-14.6)	122.7 (74.6-184.2)	122.4 (93.5-156.5)	35.7	36.9	61.2
	Iran	16.7 (13.0-20.1)	158.8 (130.4-182.6)	163.1 (136.9-181.8)	10.6 (8.2-12.6)	121.3 (98.8-138.5)	176.2 (136.4-190.3)	–20.2	37.7	98.0
	Iraq	17.4 (7.4-31.6)	298.5 (145.6-486.0)	237.4 (169.3-336.8)	8.4 (5.0-13.6)	110.8 (76.2-156.2)	106.1 (86.5-128.0)	–22.0	–24.4	–2.9
	Jordan	15.2 (8.9-24.0)	274.6 (185.5-394.6)	251.4 (200.0-334.0)	5.5 (3.1-9.3)	92.1 (59.4-139.5)	136.0 (102.9-186.6)	–25.2	3.5	44.5
	Lebanon	21.5 (12.3-35.3)	348.6 (225.5-506.7)	302.1 (239.3-374.7)	13.8 (7.9-23.6)	194.7 (124.4-285.3)	222.5 (174.9-279.2)	6.0	22.0	24.9
	Libya	24.5 (13.0-44.1)	420.5 (267.5-618.9)	340.0 (261.3-438.7)	22.0 (11.6-39.0)	373.2 (225.4-575.0)	309.4 (224.1-416.2)	1.4	95.9	73.6
	Morocco	34.5 (18.9-56.0)	717.9 (458.0-1030.1)	668.3 (488.1-820.3)	26.2 (13.1-46.9)	517.7 (306.5-812.3)	494.1 (344.1-647.7)	–11.3	1.9	31.2
	Pakistan	70.2 (40.3-115.1)	565.8 (372.8-824.1)	443.3 (352.6-543.4)	52.1 (25.5-103.3)	433.4 (255.2-704.4)	350.3 (250.5-491.3)	25.4	38.7	25.6
	Palestine	10.7 (6.5-16.3)	182.8 (119.6-261.9)	245.2 (194.7-297.8)	6.9 (3.9-11.4)	127.2 (83.3-184.1)	202.4 (153.0-245.4)	18.9	39.1	64.0
	Syria	12.0 (7.1-19.0)	173.2 (117.2-243.8)	140.7 (115.5-183.0)	8.8 (4.4-15.1)	107.1 (64.6-171.8)	123.7 (91.8-158.2)	–47.4	–7.7	58.6
	Tunisia	13.2 (7.6-20.9)	259.1 (168.2-375.4)	269.5 (216.5-339.7)	8.3 (4.2-15.4)	165.5 (95.9-259.7)	199.6 (141.2-274.6)	–32.1	–11.3	34.8
	Sudan	23.7 (9.4-43.9)	339.9 (134.9-595.9)	284.8 (173.1-430.4)	15.1 (7.2-26.8)	248.1 (113.8-415.3)	217.8 (140.3-309.9)	0.1	22.5	16.7
	Yemen	25.2 (10.3-45.3)	448.1 (192.1-769.9)	381.2 (217.9-575.2)	17.9 (8.4-30.8)	335.1 (157.4-561.7)	311.5 (208.3-450.8)	31.5	51.2	51.7
High-income countries globally		24.6 (20.6-29.7)	399.8 (357.9-445.7)	339.4 (321.9-357.8)	20.7 (16.4-26.2)	275.5 (228.7-331.6)	249.9 (224.1-278.0)	–15.4	–26.2	3.4
**High-income countries in the EMR**		7.6 (4.5-12.1)	112.0 (75.3-162.9)	154.4 (121.5-199.7)	5.3 (2.8-9.0)	76.9 (46.7-121.4)	124.9 (92.9-161.6)	28.1	111.6	90.6
	Bahrain	16.1 (8.0-25.3)	160.0 (109.8-226.1)	333.0 (271.0-424.9)	9.7 (4.7-16.3)	56.5 (37.6-83.6)	166.5 (132.9-214.7)	–5.5	5.7	86.7
	Kuwait	8.6 (5.9-12.1)	121.8 (90.5-158.9)	115.5 (100.4-131.5)	4.8 (3.0-7.3)	72.0 (52.0-99.3)	62.6 (52.7-73.8)	1.1	66.3	71.4
	Oman	14.7 (8.1-24.9)	254.5 (157.3-388.9)	268.9 (201.6-361.8)	115.1 (84.6-149.4)	72.4 (44.4-112.7)	121.4 (81.0-174.6)	17.3	28.0	40.3
	Qatar	5.4 (3.1-8.9)	72.0 (45.5-111.6)	266.4 (202.6-346.4)	2.2 (1.2-3.6)	34.7 (21.4-53.7)	135.6 (100.0-177.5)	192.8	130.9	129.8
	Saudi Arabia	6.1 (3.7-9.6)	95.8 (66.6-135.4)	111.4 (89.8-143.0)	4.4 (2.3-7.6)	82.0 (50.7-127.8)	95.0 (72.7-119.9)	41.8	111.9	68.5
	United Arab Emirates	9.1 (5.0-15.6)	113.7 (72.8-176.0)	486.3 (356.1-624.4)	6.9 (12.2-3.7)	80.4 (46.5-129.8)	354.4 (250.7-472.1)	3.7	258.9	185.6
Global average (122 countries)		65.8 (57.2-73.7)	795.3 (726.2-874.5)	596.3 (562.2-655.4)	44.6 (40.0-49.1)	631.5 (575.4-669.8)	491.6 (456.4-512.0)	–18.3	3.2	35.7
EMR average (22 countries)		29.9 (21.0-42.1)	413.6 (340.7-501.7)	349.1 (317.9-381.6)	25.2 (16.1-40.1)	299.1 (218.8-405.3)	261.2 (223.5-303.5)	–2.0	–11.8	8.0

^a^DALY: disability-adjusted life year.

^b^Relative difference (%) in the number of DALYs = ([number of cervical cancer DALYs in 2017 – number of cervical cancer DALYs in 2000]/number of cervical cancer DALYs in 2000)*100.

^c^EMR: Eastern Mediterranean Region.

## Discussion

### Principal Findings

In this study, we report the results of the data analysis on both mortality and DALYs associated with cervical cancer in 22 countries in the EMR. We found a change in the number of deaths and mortality rates from the year 2000 to the year 2017. Although the number of deaths due to cervical cancer has increased in the year 2017 (n=8537) compared to that in the year 2000 (n=6326) in the EMR, the mortality rate decreased in 2017 (2.5 per 100,000) compared to that in 2000 (2.7 per 100,000). The population expansion in the younger age groups may have contributed to the reduction in the mortality rates. For example, the population size in EMR-LICs in 2017 increased two-fold in comparison to that in the year 2000. According to the causation and nature of cervical cancer, there exists 10-20 years of gap between HPV infection and the development of cervical cancer. Thus, this disease is not seen in the younger generation; therefore, population expansion does not affect the number of deaths whereas mortality rate calculation under its influence decreases, as reported in previous studies [18]. Previous studies have shown that mortality rates and DALY rates are low in Saudi Arabia and Iran [18,19]; therefore, 1 study suggested that the cost-effectiveness of investment on HPV vaccination should be examined and if the incidence of cervical cancer is truly low, there is no reason for public health to institute either screening or vaccination [20].

Our results showed that large differences existed within the EMR in both deaths and DALYs. Considering the stratification by country income level, we found that HICs carried the largest burden of deaths and the number burden of DALYs for the 2 reference years (85.5%) compared to MICs and LICs (34.2% and 33.3%, respectively) and for all the 3 age groups (25.6%, 110%, and 74.8% vs 13.5%, 27.4%, and 39% vs 12.8%, 49.6%, and 25.1%, respectively). The EMR-HICs, unlike the HICs globally, may have higher relative difference even in comparison to LICs. This might have different causes apart from their economic level that are common in countries of the EMR.

In our study, we consider that one cause that may be relevant is the economic development of the Gulf region in the last 3 decades. This development may have improved the access to health care services, education, and changes in lifestyle and nutrition and traditional social and family structures [11]. For example, marriage in some areas includes the practice of either polygamy or temporary marriage, known by the names of “mesiar and mesfar”; this may contribute to the increase in the prevalence of HPV infections [21]. The first notable factor in this region is the lack of a reliable system of registry for diseases, including cervical cancer. According to the World Health Organization (WHO) cancer country profiles, Afghanistan [22], Somalia [23], Djibouti [24], Libya [25], and Pakistan [26] do not have a cancer registry. As per the WHO, the situation of cervical cancer in EMR should be assessed and improved [1]. The WHO’s first step is related to primary prevention through immunization against HPV of all 9-13-year-old girls before their sexual debut. HPV in almost all of the cases is the main attributable risk factor for cervical cancer, and the risk of developing cervical cancer is highly associated with the age of first intercourse; the younger the individual, the more is the probability of developing infection into papillomavirus-associated cancer [27]. All the countries in the EMR report the prevalence of HPV strains 16 and 18 (the most oncogenic subtypes of HPV) in women with normal cytology—from 0.2% in Kuwait [28] up to 4.8% in Somalia [29] and Djibouti [30], which has the potential to progress to cervical precancer and cancer in 10-20 years. The prevalence of HPV strains 16 and 18 in low-grade lesions was from 20.8% in Morocco [31] and Libya [32] up to 42.9% in Iran [33] and that in high-grade lesions was from 45.7% in Somalia [29] and Djibouti [30] up to 63.4% in Afghanistan [34] and Pakistan [35]. In women with cervical cancer, the prevalence was from 58.6% in Iran [33] up to 92.7% in Jordan [36]. This shows the importance of HPV vaccination in these countries to ensure that the infection does not progress to cervical precancer and cancer. Among the EMR countries, Libya [31] has had a national HPV vaccination program since 2013, and Emirates [37] had a partial program since 2008. In addition, in the EMR, education and knowledge on cervical cancer are reported as important determinative factors in primary prevention [38]. The increased education of women and their families about cervical cancer, its causes, signs, symptoms, and preventions will engage them more in the preventive and screening practices [38]. The WHO’s other primary preventive measures are “Girls and boys, as appropriate—Health information and warnings about tobacco use—Sexuality education tailored to age and culture—Condom promotion/provision for those engaged in sexual activity—Male circumcision” [1]. Almost all of the countries of the EMR report more than 80% male circumcision, and the usage of condom is low from 0.0% in Somalia [29] and Sudan [39] up to 13.8% in Iran [33]. With regard to the sexual education for girls and boys, a study conducted in Iran generalized the matter to all Muslim countries and reported the social unacceptability of sexual health education for unmarried people due to religious and cultural prohibition of extramarital sex, in particular, for girls. Further, sexual education highlights the effect of technology improvement and internet on the sexual behaviors of youth, including extramarital relationships, and this should not be denied [40]. The WHO’s second step to comprehensive prevention and control of cervical cancer is related to secondary prevention through effective screening of women older than 30 years and treatment as needed with low-cost technology, for example, visual inspection with acetic acid (VIA) followed by cryotherapy [1]. In the EMR, Somalia [29], Djibouti [30], Libya [32], Sudan [39], and Yemen [41] have no cervical cancer screening programs. Two countries, Tunisia [42] and Morocco [31], have quality assurance structures, which supervise and monitor the screening; however, none of them has an active invitation for screening. In Egypt, the cost of screening and treatment is not covered by public funding; therefore, this is considered as a nonaffordable service by over 40% of the Egyptian women [43]. Cytology is considered as the main screening test in EMR, except for Morocco [31] and Pakistan [35] where VIA is used. The VIA is suggested especially in LICs and rural areas that do not have access to health care facilities [44]. Further, women face additional challenges such as language, transportation, insurance, and family pressure as inhibitors for obtaining regular cervical cancer screening, especially in case it is perceived as a threat to their cultural and religious values [45]. The reported barriers in the rural areas are the financial costs, acceptance by husbands, and availability of a female health care provider [43].

The early detection of cancer reduces financial burdens, both on the health care system and economically [46]. Screening and vaccination programs are considered to be more accessible and effective than relying only on cancer treatment. For example, in Saudi Arabia, the pap test is fully subsidized in public hospitals, and it costs US $50 in private clinics. Yet, these costs are negligible compared to the costs of cervical cancer treatment, where it can vary between US $14,000-US $30,000 during the first year of therapy [21].

The WHO recommends a comprehensive prevention and control of cervical cancer, which is related to tertiary prevention for women older than 30 years. This includes treating invasive cancer at any age, including ablative surgery, radiotherapy, and chemotherapy palliative care [1]. However, radiotherapy is not generally available in Afghanistan [22], Somalia [23], Djibouti [24], Egypt [47], and Pakistan [26] and is generally available in other countries of the EMR. The availability of radiotherapy centers ranges from 1 center in Yemen [48], Bahrain [49], Kuwait [50], Oman [51], and Qatar [52] up to 40 centers in Iran [53]. The availability of radiation clinics ranges from 3 centers in Qatar [52] up to 237 centers in Egypt [47]. Chemotherapy is not available in Afghanistan [22], Somalia [23], Djibouti [24], Egypt [47], and Pakistan [26], but it is available in other countries of the EMR. Finally, community/home care for people with advanced-stage cancer is only available in 2 countries of the EMR, namely, Bahrain [49] and Kuwait [50].

### Study Limitations

The ongoing civil unrest may have affected the quality of the health data in the EMR, as the GBD study uses modelling techniques to generate the estimates based on other available variables or data from countries with a similar health profile in the neighboring region for countries that have insufficient data. In this study, we used data on the numbers of mortality, crude death rate, and DALY rate. Moreover, the incidence of cervical cancer was not examined.

### Conclusion

Cervical cancer is becoming a major problem in the EMR, and its burden might increase due to the population growth. There is a need for effective interventions to reduce the burden of cervical cancer. This includes prevention measures such as HPV vaccination, early detection of cervical cancer, and reducing the risk factors associated with cervical cancer.

## Abbreviations

DALY: disability-adjusted life year
EMR: Eastern Mediterranean Region
GBD: global burden of disease
HIC: high-income country
HPV: human papillomavirus
LIC: low-income country
LMICs: low- and middle-income countries
MIC: middle-income country
VIA: visual inspection with acetic acid
WHO: World Health Organization
